# Selective cytotoxicity of epidithiodiketopiperazine DC1149B, produced by marine-derived *Trichoderma lixii* on the cancer cells adapted to glucose starvation

**DOI:** 10.1007/s11418-019-01357-w

**Published:** 2019-08-21

**Authors:** Rui Tang, Atsushi Kimishima, Ryosuke Ishida, Andi Setiawan, Masayoshi Arai

**Affiliations:** 1 Graduate School of Pharmaceutical Sciences, Osaka University, Yamadaoka 1-6, Suita, Osaka 565-0871 Japan; 2 Department of Chemistry, Faculty of Science, Lampung University, Jl. Prof. Dr. Sumantri Brodjonegoro No. 1, Bandar Lampung, 35145 Indonesia

**Keywords:** Epidithiodiketopiperazine, DC1149B, Marine-derived *Trichoderma lixii*, Cancer, Glucose starvation

## Abstract

The core of solid tumors is characterized by hypoxia and a nutrient-starved microenvironment and has gained much attention as targets of anti-cancer drugs. In the course of search for selective growth inhibitors against the cancer cells adapted to nutrient starvation, epidithiodiketopiperazine DC1149B (**1**) together with structurally related compounds, trichodermamide A (**2**) and aspergillazine A (**3**), were isolated from culture extract of marine-derived *Trichoderma lixii*. Compounds **1** exhibited potent selective cytotoxic activity against human pancreatic carcinoma PANC-1 cells cultured under glucose-starved conditions with IC_50_ values of 0.02 µM. The selective index of the compound **1** was found to be 35,500-fold higher for cells cultured under glucose-starved conditions than those under the general culture conditions. The mechanistic analysis indicated that compound **1** inhibited the response of the ER stress signaling. In addition, these effects of compound **1** could be mediated by inhibiting complex II in the mitochondrial electron transport chain.

## Introduction

Solid tumors contain hypoxic and nutrient-starved regions due to the abnormal cell proliferation coupled with the defective and disorganized vascular supply [1]. The cancer cells that have adapted to this tumor microenvironment are assumed to develop aggressive phenotype, impaired angiogenesis, and drug resistance [2, 3]. Since the hypoxic and nutrient-starved tumor microenvironment differs significantly from the normal tissues, the compounds with selective cytotoxicity against the cancer cells in tumor microenvironment would have great therapeutic potential.

The marine flora and fauna are a rich source of therapeutic drugs because of their chemical and biological diversity. Some of the marine-derived compounds have been reported to inhibit the growth of cancer cells adapted to the hypoxic or nutrient-starved conditions. For example, furospinosulin-1 (furanosesterterpene) and dictyoceratins-A and -C (sesquiterpene phenols) isolated from the marine sponge *Dactylospongia elegans* were shown to exhibit the selective growth inhibitory activities against the hypoxia-adapted human prostate cancer cell line DU145 [4, 5]. Recently, we reported the cytotoxic activities of polybrominated diphenyl ethers, *N*-methylniphatin A (new 3-alkyl pyridine alkaloid) and biakamides (unique new polyketides) isolated from Indonesian marine sponges of *Dysidea* sp., *Xestospongia* sp., and *Petrosaspongia* sp., respectively, against human pancreatic carcinoma cell line PANC-1 adapted to glucose-deficient growth conditions [6–8]. In this study, we present the isolation of epidithiodiketopiperazine DC1149B (**1**), and structurally related but inactive compounds, trichodermamide A (**2**) and aspergillazine A (**3**) isolated from the culture extract of marine-derived *Trichoderma lixii*., and the cytotoxic activity of compound **1** on nutrient-starved cancer cells, and propose the plausible mechanism of its action.

## Materials and methods

### Materials

Dulbecco’s Modified Eagle’s medium (DMEM), WST-8 colorimetric reagent, and KCN were purchased from Nacalai Tesque, Inc. (Kyoto, Japan). Fetal bovine serum (FBS) and Dialyzed FBS were purchased from Equitech-Bio Inc. (Kerrville, TX, USA) and Thermo Fisher Scientific Inc. (Waltham, MA, USA), respectively. Anti-Akt, Anti-phosphorylated Akt, anti-GRP78, and anti-β-actin antibodies were obtained from Cell Signaling Technology, Inc. (Danvers, MA, USA). Horseradish peroxidase (HRP)-linked anti-rabbit IgG antibody (GE Healthcare Life Sciences, Buckinghamshire, UK) was used as secondary antibody. Mito Check Complex Activity Assay Kit (Cayman Chemical, Ann Arbor, MI, USA) was used to evaluate the effect of compound **1** on the mitochondrial complex I–V. Oxygen consumption of cells was measured by Oxygen Consumption Rate (OCR) Assay Kit (Cayman Chemical, Ann Arbor, MI, USA). Rotenone, thenoyltrifluoroacetone (TTFA), carbonyl cyanide *m*-chlorophenylhydrazone (CCCP), antimycin A, and oligomycin A were obtained from Tokyo Chemical Industry Co., LTD. (Tokyo, Japan), Wako Pure Chemical Industries, Ltd. (Osaka, Japan), Sigma-Aldrich (St. Louis, MO, USA), LKT Laboratories, Inc. (St. Paul, MN, USA), and Cayman Chemical (Ann Arbor, MI, USA), respectively. Other chemicals were purchased from Sigma-Aldrich (St. Louis, MO, USA) or Kishida Chemical Co., Ltd. (Osaka, Japan).

### Isolation of compounds **1**–**3**

The marine-derived fungus 15G49-1 was isolated from an unidentified marine sponge collected at Mentawai, Indonesia in 2015. The strain was identified as *Trichoderma lixii* by Techno Suruga Laboratory Co., Ltd. (Shizuoka, Japan) based on the morphology and 5.8S rDNA sequence. The *Trichoderma lixii* 15G49-1 was cultured in the rice medium (totally 500 g of unpolished rice and 1000 ml of artificial sea water) under static condition at 30 °C for 2 weeks. The culture was extracted by acetone and a mixed organic solvent of acetone/MeOH/EtOAc (4:2:1), followed by combining and evaporating the organic solvents under reduced pressure to obtain a crude extract. The extract was partitioned into a water/EtOAc mixture. The active EtOAc soluble portion (13.6 g) was further partitioned into an *n*-hexane/90% aq. MeOH mixture. On the guidance of bioassay, the 90% MeOH-soluble portion [8.7 g, IC_50_ (Glucose Deficient Medium) = 9.0 µg/ml, IC_50_ (General Glucose Medium) = > 100 µg/ml] was fractionated by silica gel column chromatography [eluted with *n*-hexane:EtOAc] to obtain seven fractions (Fr.M1 ~ Fr.M7). Among these fractions, the Fr.M3 (1.2 g, eluted with *n*-hexane:EtOAc = 1:5) showed selective growth inhibition on the PANC-1 cells adapted to glucose starvation [IC_50_ (Glucose Deficient Medium) = 2.5 µg/ml, IC_50_ (General Glucose Medium) = > 100 µg/ml]. The active Fr.M3 was then separated by open ODS column chromatography (eluted with MeOH:H_2_O) to obtain six fractions (Fr.M3-1 ~ Fr.M3-6). The active Fr.M3-3 [289 mg, eluted with MeOH:H_2_O = 2:3, IC_50_ (Glucose Deficient Medium) = 0.3 µg/ml, IC_50_ (General Glucose Medium) = > 100 µg/ml] was further purified by reversed-phase HPLC [Cosmosil 5C_18_-MS-II (10 mm id × 250 mm); eluted with MeOH:H_2_O = 1:1] to give DC1149B (**1**, 39 mg) [9, 10], trichodermamide A (**2**, 9.5 mg) [11, 12] and aspergillazine A (**3**) containing fraction (Fr.M3-3-3, 46 mg). The Fr. M3-3-3 was recrystallized with CHCl_3_/MeOH (3:1) to give aspergillazine A (**3**, 10.3 mg) [12, 13]. Compounds **1**–**3** were identified using ESI–TOF–MS and NMR analyses, and comparison with authentic spectral data [9–13].

### Cell culture and bioassay

Human pancreatic carcinoma cell line PANC-1 was maintained in the DMEM supplemented with heat-inactivated 10% FBS and kanamycin (50 µg/ml) in a humidified atmosphere with 5% CO_2_ at 37 °C. To induce nutrient starvation, PANC-1 cells were cultured in the Glucose Deficient Medium [Basal Medium (25 mM HEPES buffer (pH 7.4) supplemented with 6.4 g/l NaCl, 700 mg/l NaHCO_3_, 400 mg/l KCl, 265 mg/l CaCl_2_·2H_2_O, 200 mg/l MgSO_4_·7H_2_O, 125 mg/l NaH_2_PO_4_, 0.1 mg/l Fe(NO_3_)·9H_2_O, 15 mg/l Phenol red, 10 mL/l MEM vitamin solution (X100) (GIBCO, Carlsbad, CA), 200 mmol/l l-glutamine solution (GIBCO, Carlsbad, CA), and 25 mg/l kanamycin) containing 10% dialyzed FBS]. The General Glucose Medium [Basal Medium supplemented with 10% FBS and 2.0 g/l glucose (final 25 mM)] was also used for control cells.

The bioassay was carried out according to a method described previously [6]. Briefly, PANC-1 cells (1 × 10^4^ cells/100 µl in 96 well plastic plate) were pre-incubated in the DMEM supplemented with 10% FBS for 24 h. The medium was then replaced with either the General Glucose Medium or Glucose Deficient Medium to induce cells adaption to the nutrient starvation. After 12-h incubation, the serially diluted samples were added, and the cells were incubated for an additional 12 h in a humidified atmosphere with 5% CO_2_ at 37 °C. The cell proliferation was detected using the WST-8 colorimetric reagent. The IC_50_ value was determined by linear interpolation from the growth inhibition curve. We assessed the selectivity of the cytotoxic activity (Selective Index, S.I.) based on the difference in the IC_50_ values obtained for experiments with General Glucose Media and Glucose Deficient Media.

### Western blotting analysis

PANC-1 cells (3 × 10^5^ cells/2 ml in 6-well plastic plate) were pre-incubated in the DMEM supplement with 10% FBS for 24 h. The medium was then replaced with either General Glucose or Glucose Deficient Medium. After 12-h incubation, compound **1** (0.02–0.3 µM) or antimycin A (0.3 nM, positive control) was added to each well and the cells were incubated for an additional 12 h in a humidified atmosphere with 5% CO_2_ at 37 °C. Subsequently, the cells were rinsed with ice-cold PBS and lysed in the lysis buffer [50 mM Tris–HCl (pH 8.0) containing 150 mM NaCl, 5 mM EDTA, 1% glycerol, 1% NP-40, 1% protease inhibitor cocktail, and 1% phosphatase inhibitor cocktail]. The cell lysate was analyzed by SDS-PAGE and transferred onto PVDF membranes (GE Healthcare Life Sciences Buckinghamshire, UK). The membranes were then incubated with appropriate primary antibodies followed by HRP-conjugated secondary antibodies, and the immunopositive bands were visualized using an ECL kit (GE Healthcare Life Sciences). The luminescent signals were analyzed using an Image Quant LAS4010 Scanner (GE Healthcare Life Sciences).

### Effects of compound 1 on the oxygen consumption of PANC-1 cells

Oxygen consumption rate of PANC-1 cells was assayed using OCR Assay Kit (Cayman Chemical, Ann Arbor, MI, USA) as per the manufacturer’s instructions. Briefly, pre-cultured PANC-1 cells (8.0 × 10^4^ cells) in the black, clear bottom 96-well plate (Corning Incorporated, NY, USA) were incubated with General Glucose Medium for 18 h at 37 °C. The cells were replenished with 140 µl of fresh medium, and the test compound was added. This was followed by adding the phosphorescent probe to measure the oxygen consumption. 100 µl of mineral oil was added in each well to restrict oxygen diffusion. The signals were measured by an Infinite M1000 microplate reader (Tecan Group Ltd., Mannedorf, Switzerland) using time-resolved mode at Ex 380 nm and Em 650 nm for 180 min with 1-min time interval. The linear regression was applied after subtracting the blank, and the oxygen consumption rate was indicated by the slope of each signal profile.

### Statistical analysis

Data are shown as means ± standard errors of *n* = 3 independent experiments, and the differences between data sets were assessed by Dunnett’s test. Differences with *p* values of less than 0.05 were considered significant.

## Results and discussion

### Cytotoxic activity of compounds **1**–**3** against the PANC-1 cells cultured under both glucose-deficient conditions and general culture conditions

**Fig. 1:**
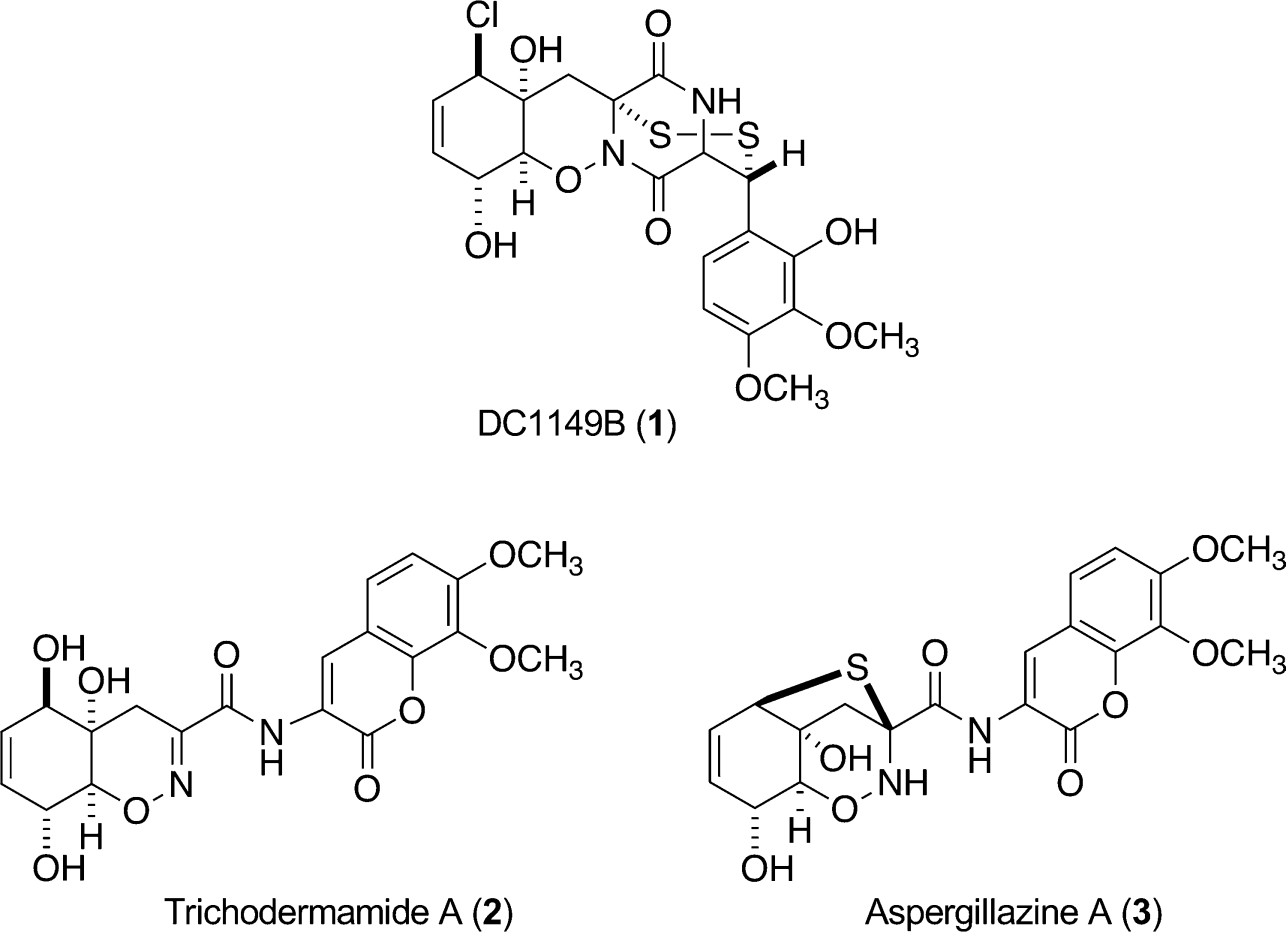
Chemical structures of compounds **1**–**3**

The bioassay-guided separation of the active 90% MeOH-soluble portion from culture extract of marine-derived *Trichoderma lixii* 15G49-1 led to the isolation of DC1149B (**1**), trichodermamide A (**2**), and aspergillazine A (**3**) (Fig. 1). We then evaluated the cytotoxic activities of compounds **1**–**3** against the PANC-1 cells cultured under both glucose-deficient and general culture conditions. Antimycin A, which inhibits the growth of PANC-1 cells adapted to the nutrient-starved conditions, was used as the positive control [14].

As shown in Table 1, DC1149B (**1**) showed a higher cytotoxic activity against the PANC-1 cells adapted to glucose starvation (IC_50_ = 0.02 µM) when compared with cells cultured with General Glucose Medium (IC_50_ = 710 µM). The S. I. value of compound **1** was evaluated to be more than 35,000. On the other hand, the cytotoxic activities of trichodermamide A (**2**) and aspergillazine A (**3**) were very weak under glucose-starved conditions. These results indicated that compounds **1** had selective cytotoxic activity against the PANC-1 cells adapted to nutrient starvation.

**Table 1:** Cytotoxic activity of compounds **1**–**3** against PANC-1 cells under the glucose-deficient and general culture conditions

	IC_50_ (µM)		
	Glucose (−)^a^	Glucose (+)^b^	S.I.^c^
**1**	0.02	710	35,500
**2**	270	> 1000	> 3.7
**3**	> 1000	> 1000	> 1.0
Antimycin A^d^	0.0003	288	960,000

^a^Conditions of glucose deficient medium

^b^Conditions of general glucose medium

^c^Selective index

^d^Compound for positive control

DC1149B (**1**) was first reported as a secondary metabolite of *Trichoderma longibrachiatum* and showed weak cytotoxic activity against rat adrenal pheochromocytoma cell line PC12 (IC_50_ = 21.9 µg/ml, 42.4 µM) and human cervix epithelioid carcinoma HeLa cells (IC_50_ = 50.1 µg/ml, 97.1 µM) [9]. H. Yamazaki et al. also showed that compound **1** exhibited moderate cytotoxicity on the human leukemic Jurkat cell line with IC_50_ value of 5.1 µM [10]. For the first time, DC1149B (**1**) was shown to exhibit the preferential cytotoxic activity against PANC-1 cells adapted to glucose starvation. We next explored the underlying mechanism of DC1149B (**1**) cytotoxicity.

### Effects of compound 1 on the Akt signaling and induction of GRP78

**Fig. 2:**
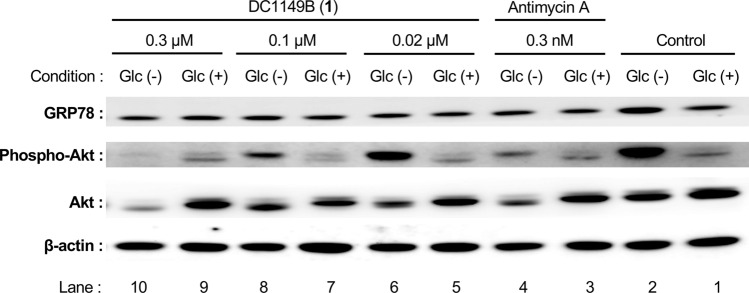
Effects of compound **1** on the Akt signaling and induction of GRP78. Glc (−): Culture in the presence of Glucose Deficient Medium. Glc (+): Culture in presence of General Glucose Medium. The PANC-1 cells cultured in the Glucose Deficient Medium were treated with indicated concentration of compound **1**. Cell lysate was resolved by using SDS-PAGE and detected with antibodies against the indicated proteins

Recent studies on cancer cells adapted to nutrient starvation have revealed that the activation of PI3k/Akt/mTOR signaling pathway and the unfolded protein response (UPR) such as induction of glucose-regulated protein 78 (GRP78) were important for the adaptation of cancer cells to nutrient starvation [15, 16]. Therefore, these processes have attracted much attention as possible drug targets for cancer chemotherapy. This observation prompted us to investigate the effect of compound **1** on the Akt signaling and the induction of GRP78 by utilizing a western blotting method (Fig. 2). Antimycin A was used as a positive control for comparison. The PANC-1 cells cultured in the Glucose Deficient Medium increased the expression levels of phosphorylated Akt and GRP78 proteins compared with cells cultured in the General Glucose Medium (Fig. 2, lanes 1 and 2). Treatment with DC1149B (**1**) at the lowest test concentration of 0.02 µM decreased the expression of GRP78 in the nutrient-starved cells to the basal level of cells cultured in General Glucose Medium (Fig. 2, lanes 5–10). Also, treatment with DC1149B (**1**) reduced the levels of Akt and phosphorylated Akt in the nutrient-starved cells in a dose-dependent manner (Fig. 2, lanes 5–10). These results indicated that DC1149B (**1**) inhibited response of ER stress signaling. Besides, compound **1** exhibited similar effects with antimycin A on the PANC-1 cells adapted to the nutrient-starved conditions (Fig. 2, lanes 3 and 4). The present results, therefore, suggest that compound **1** showed a similar behavior with that of antimycin A against the PANC-1 cells cultured in the Glucose Deficient Medium.

### Effect of compound **1** on the mitochondrial electron transport chain

**Fig. 3:**
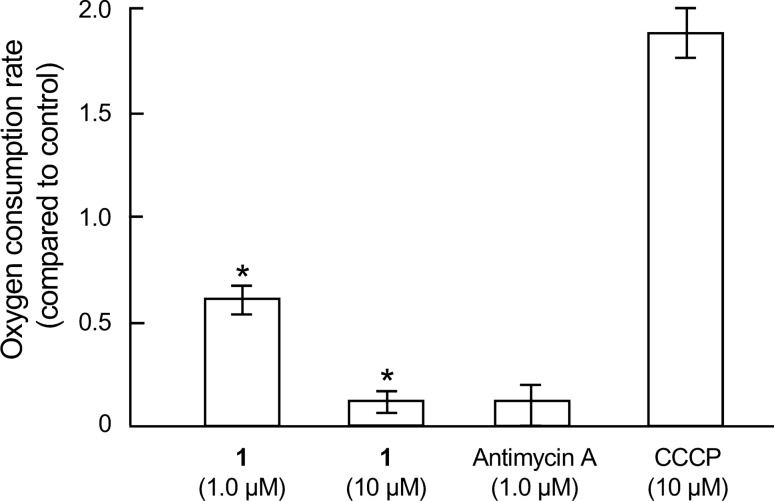
Effect of compound **1** on the oxygen consumption of PANC-1 cells. Pre-cultured PANC-1 cells (8.0 × 10^4^ cells) in the 96-well plate was incubated in General Glucose Medium for 12 h at 37 °C. The medium was then replaced with each fresh medium, and the test compound was added followed by adding the phosphorescent probe to measure the oxygen consumption. The signals were measured by a Tecan infinite M1000 using time-resolved mode. Differences were considered significant at *p < 0.05

Antimycin A is a known inhibitor of complex III in the mitochondrial electron transport chain [17]. Therefore, we next examined the effect of compound **1** on the function of mitochondrial complex I–V using the Mito Check Complex Activity Assay Kit. As shown in Table 2, compound **1** inhibited complex II with IC_50_ values of 19 µM, while other complexes remained unaffected up to 30 µM. It is known that inhibitors of the mitochondrial electron transport chain affect the oxygen consumption of the treated cells [18]. Since the inhibitory effect of compound **1** on the complex II was weak compared with that on the PANC-1 cells adapted to glucose-starved conditions (Table 1), we explored whether DC1149B (**1**) inhibited the oxygen consumption on the PANC-1 cells (Fig. 3). As a result, positive control of Antimycin A inhibited the oxygen consumption on the PANC-1 cells cultured in the General Glucose Medium, while the carbonyl cyanide *m*-chlorophenylhydrazone (CCCP, negative control), which is uncoupling substance of mitochondria, showed the opposite effect to antimycin A [18]. Compound **1** also inhibited the oxygen consumption on the PANC-1 cells under general culture conditions (Fig. 3). This result indicated that compound **1** inhibits complex II in the mitochondrial electron transport chain. We also evaluated the effect of compound **1** on the oxygen consumption of PANC-1 cells cultured in the Glucose Deficient Medium. However, the oxygen consumption rate of PANC-1 cells could not be determined, as the oxygen consumption of PANC-1 cells in the Glucose Deficient Medium was significantly lower than that in the General Glucose Medium. Collectively, our results indicate that the selective cytotoxicity of compound **1** might be mediated by the inhibition of complex II in the mitochondrial electron transport chain, while further study is necessary to consider whether complex II is major target for DC1149B (**1**) as a selective inhibitor against cancer cells adapted to glucose-starved conditions.

**Table 2:** Effect of compound **1** on the mitochondrial electron transport chain

	IC_50_ (µM)				
	Complex I	Complex II	Complex II/III	Complex IV	Complex V
Compound **1**	> 30	19	> 30	> 30	> 30
Positive control^a^	0.11	65	0.03	7.7	0.36

^a^Compounds used as a positive control are rotenone, thenoyltrifluoroacetone, antimycin A, KCN, and oligomycin A for complex I, II, III, IV, and V, respectively

## Conclusions

In our search for inhibitors against the cancer cells adapted to nutrient starvation, the epidithiodiketopiperazine DC1149B (**1**) was isolated from culture extract of marine-derived *Trichoderma lixii*. DC1149B (**1**) showed potent selective cytotoxicity against the cancer cells adapted to the glucose-starved conditions, with a S.I. value of 35,500. This study also explains the previously unknown mechanism of action of compound **1** on the cancer cells. Our results revealed that compound **1** inhibited the ER stress signaling, and that these effects of compound **1** could be mediated by the inhibition of complex II in the mitochondrial electron transport chain. Currently, research is underway to synthesize a probe to identify the major target of compound **1** in PANC-1 cells adapted to nutrient-starved conditions, and to observe the distribution of compound **1** in PANC-1 cells.
