# Correction to: Selective cytotoxicity of epidithiodiketopiperazine DC1149B, produced by marine-derived *Trichoderma lixii* on the cancer cells adapted to glucose starvation

**DOI:** 10.1007/s11418-021-01499-w

**Published:** 2021-03-11

**Authors:** Rui Tang, Atsushi Kimishima, Ryosuke Ishida, Andi Setiawan, Masayoshi Arai

**Affiliations:** 1 Graduate School of Pharmaceutical Sciences, Osaka University, Yamadaoka 1-6, Suita, Osaka 565-0871 Japan; 2 Department of Chemistry, Faculty of Science, Lampung University, Jl. Prof. Dr. Sumantri Brodjonegoro No. 1, Bandar Lampung, 35145 Indonesia

## Correction to: Journal of Natural Medicines (2020) 74:153–158 10.1007/s11418-019-01357-w

The article Selective cytotoxicity of epidithiodiketopiperazine DC1149B, produced by marine-derived *Trichoderma lixii* on the cancer cells adapted to glucose starvation, written by Rui Tang, Atsushi Kimishima, Ryosuke Ishida, Andi Setiawan and Masayoshi Arai, was originally published Online First without Open Access. After publication in volume 74, issue 1, page 153–158 the author decided to opt for Open Choice and to make the article an Open Access publication. Therefore, the copyright of the article has been changed to © The Author(s) 2021 and the article is forthwith distributed under the terms of the Creative Commons Attribution 4.0 International License (https://creativecommons.org/licenses/by/4.0/), which permits use, sharing, adaptation, distribution and reproduction in any medium or format, as long as you give appropriate credit to the original author(s) and the source, provide a link to the Creative Commons license, and indicate if changes were made.

The original article has been updated.

